# Cardiac sparing characteristics of internal mammary chain radiotherapy using deep inspiration breath hold for left-sided breast cancer

**DOI:** 10.1186/s13014-018-1052-8

**Published:** 2018-05-31

**Authors:** Macklin H. Nguyen, Myra Lavilla, Janice N. Kim, L. Christine Fang

**Affiliations:** 1 School of Medicine, University of Washington, 1959 NE Pacific Street, Seattle, WA 98195 USA; 2 Seattle Cancer Care Alliance, 825 Eastlake Avenue East, Seattle, WA 98109 USA; 3 Department of Radiation Oncology, University of Washington, 1959 NE Pacific Street, Seattle, WA 98195 USA

**Keywords:** Deep inspiration breath hold, Breast cancer, Internal mammary nodes, Regional nodal irradiation, Cardiac sparing, Dosimetry

## Abstract

**Background:**

While breast radiotherapy typically includes regional nodal basins, the treatment of the internal mammary nodes (IMN) has been controversial due to concern for long-term cardiac toxicity. For high risk patients where IMN treatment is warranted, there is limited data with regards to the degree of heart sparing conferred by modern techniques. In this study, we sought to analyze the specific heart sparing metrics conferred by deep inspiration breath hold (DIBH) in the setting of IMN irradiation.

**Methods:**

From 2012 to 2015, 168 consecutive patients were treated with adjuvant left-sided radiotherapy using DIBH. Retrospective review identified 49 patients who received nodal irradiation, either to a supraclavicular field (SCF) and IMN (16), or to the SCF alone (33). Cardiac mean dose and dose volumes were calculated from free breathing (FB) and DIBH treatment plans, and compared by Wilcoxon signed-rank and Mann–Whitney *U* tests.

**Results:**

DIBH achieved significant reductions in mean heart dose (*p* < 0.001) in both the IMN treated group from 6.73 Gy to 2.79 Gy (− 56.4%) and the IMN untreated group from 4.77 Gy to 1.55 Gy (− 63.7%). There was a 7.3% difference in relative reduction that was not statistically significant (*p* = 0.216). Relative reductions in heart dose volume measures were all significantly lower for IMN-irradiated patients (*p* ≤ 0.012), with the greatest deficits at V_5_ that gradually diminish with increasing dose (V_25_).

**Conclusions:**

The relative heart sparing benefits of the DIBH technique are retained even with IMN inclusion. However, the addition of IMN irradiation is associated with an intrinsically greater heart dose, which translates to an estimated 9.2% proportional increase in the risk of a subsequent major coronary event. In the setting of effective cardiac sparing techniques, clinicians should take these considerations into account to guide when IMN treatment is warranted.

## Background

Radiotherapy is a key component of breast cancer treatment and often includes radiation to the draining lymph node basins in addition to the primary site. Radiation treatment following surgery greatly reduces locoregional recurrence and provides a significant improvement in long-term breast cancer mortality compared to surgery alone [1–3]. Among those who stand to benefit most are node-positive patients and a subset of node-negative patients with high risk traits such as young age, higher tumor grade, and larger size [2]. Similar benefits to locoregional recurrence, relapse free survival, and breast cancer free survival have been observed in patients who underwent radiotherapy in addition to adjuvant chemotherapy [4]. While radiation therapy has demonstrated a vital role in breast cancer treatment, there remains a lack of clarity in regards to the subset of patients in whom the treatment of internal mammary nodes (IMN) is necessary.

Uncertainty over treating the IMN stems from early surgical studies that suggested no improvement in overall survival with extended radical mastectomy (radical mastectomy with IMN dissection) compared to radical mastectomy alone [5–7]. However, these conclusions may have limited clinical applicability today, considering that extended radical mastectomy is in itself a morbid procedure and that patients included in aforementioned surgical series did not receive adjuvant radiotherapy or systemic therapy [8]. Additionally, one of these studies [5] observed a significant reduction in locoregional recurrence at 10 years among patients who received extended radical mastectomy (14% versus 24%), a finding that suggests that there may be benefits to targeting the IMN that have not been fully elucidated.

Ambivalence towards IMN irradiation also derives from their proximity to the heart and concern for long-term cardiac toxicity. One technique that reduces cardiac exposure is deep inspiration breath hold (DIBH). During the delivery of radiotherapy, DIBH promotes greater separation between the heart and target volume including the IMN, and allows for significant decreases in in-field cardiac volume, mean cardiac dose, and dose volume metrics compared to free breathing (FB) [9, 10]. Two predominant modalities for respiratory gating include spirometry-based active breathing control (ABC) [11] and video-based real-time position management (RPM) [12]. More recently, surface imaging has emerged as another viable technique. At our institution, we utilize electromagnetic Beacon® transponders by Varian Medical Systems (Palo Alto, California, USA). Originally used for monitoring intra-fraction motion and position in prostate radiotherapy [13], this system has shown comparable feasibility and accuracy in the setting of breast irradiation [14, 15].

In this study, we will investigate the heart sparing merits of DIBH in patients with left-sided disease, with specific consideration to the effects of IMN treatment. In light of the recent literature [8, 16–19] that has placed renewed value on IMN irradiation, we seek to quantify the degree of cardiac exposure and sparing in patients whose treatments have historically been viewed as unfavorable.

## Methods

From the 168 consecutive women with a diagnosis of primary left-sided breast cancer who received adjuvant radiotherapy with breath hold following mastectomy or breast-conserving surgery between January 2012 and February 2015, we performed a retrospective chart review and identified 49 patients who received some form of regional nodal irradiation. Of these, 33 patients received treatment to just a supraclavicular field (SCF) and 16 patients received IMN irradiation in addition to a SCF. The former cohort of 33 was used in our analysis as a comparison group. All patients were above the age of 18 and were treated at our institution. Chemotherapy was delivered at the discretion of the medical oncologist but was largely in node-positive patients or those with an intermediate to high 21-gene recurrence assay score. Local institutional review board approval was obtained to perform this analysis.

All patients had 3-D conformal radiotherapy planned using Pinnacle or CMS planning software. CTV was defined as the lumpectomy cavity with a 1 cm expansion, and PTV was CTV with a 5 mm expansion. Both CTV and PTV excluded tissue within 5 mm of the skin surface as well as the chest wall/lung interface. SCF and IMN coverage follows the Radiation Therapy Oncology Group (RTOG) atlas/guidelines, with the IMN treatment field including interspaces one to three. Patients were treated with standard fractionation at 1.8–2.0 Gy per fraction. The intact breast/chest wall was treated with opposed photon tangent beams. Partially wide tangent fields were used to accommodate patient anatomy. IMN coverage was either included in a tangent beam or a matching enface electron field. Target coverage to the target volumes were V95 > 95%, with a minimum acceptable of V90 > 90%. Mean heart dose was as low as reasonably achievable but with a maximum of 4 Gy. Additional constraints included ipsilateral lung V_20_ < 38%.

DIBH was utilized in all patients for treatment of the primary tumor bed. For daily treatment setup, the vertical displacement measured on the free breathing and breath hold simulation CT scans was used to achieve the proper volume of breath hold. A ceramic BB was placed on the sternal tattoo during CT simulation. This BB is present and can be visualized on both DIBH and FB imaging. The superior to inferior displacement of the BB was measured from the DIBH images and used for daily patient setup. Using this displacement, the radiation therapist marked the patient at the measured distance. The patient is then instructed to breathe in and asked to stop when the lasers match the mark. Digitally reconstructed radiographs (DRR) are taken to verify that the DIBH fields are appropriate and match the plan. The electromagnetic system was then used to monitor and capture daily infra-fraction stability of the breath hold using two surface transponders arranged in an “L” shape that were affixed to the chest surface, 1 cm lateral and 2 cm inferior to the isocenter. Electromagnetic beacons are tracked using non-ionizing radiofrequency that are detected with an external electromagnetic array in conjunction with three ceiling-mounted infrared cameras. The system offers real-time motion monitoring and alerts the therapist when chest excursion is outside of the predetermined therapeutic range of ±3 mm. If a patient drifts from the appropriate level of chest excursion, a visual alert cues the therapist to manually pause the treatment beam. Auditory feedback is then given, and treatment is restarted once she is able to perform another breath hold. All patients were in a supine position with both arms up and immobilized using thermal-setting foam to ensure reproducibility.

Demographic, clinical, and treatment characteristics were gathered from the electronic medical records and treatment planning systems. Treatment planning metrics including cardiac mean dose and dose volumes (V_5_-V_25_) were analyzed using SPSS Statistics 20 (IBM Corporation, Armonk, New York, USA). Percent relative reductions were calculated by dividing difference between FB and DIBH by FB irradiated heart volume. Nonparametric tests were utilized. Wilcoxon signed-rank tests were employed for dependent samples such as within patient comparisons (i.e. DIBH versus FB plans), while independent samples were compared by Mann–Whitney *U* tests (i.e. between treatment groups). Two-tailed *p*-values ≤0.05 were deemed significant.

## Results

Overall patient and tumor characteristics were similar between IMN treated and untreated groups (Table 1), with the median age at diagnosis of 47.4 years and 48 years, respectively. All patients were American Joint Committee on Cancer (AJCC) stage II or III. The IMN treated group tended to have more locally advanced disease, with a greater proportion of stage III breast cancer at 68.8% (11/16) compared to 36.4% (12/33). Receptor status was comparable between both patient cohorts.

**Table 1:** Patient demographic, disease, and treatment characteristics

	IMN Treated	IMN Untreated
Patient Characteristics		
Total (n)	16	33
Median Age (Range), years	47.4 (36.9–69.9)	48 (26.5–69.1)
Disease Characteristics		
Stage		
II	5 (31.3%)	21 (63.6%)
III	11 (68.8%)	12 (36.4%)
Node Positive	12 (75%)	25 (75.8%)
Cell Type		
Ductal	12 (75%)	30 (90.9%)
Lobular	4 (25%)	3 (9.1%)
Receptor Status		
ER+	13 (81.3%)	28 (84.8%)
PR+	12 (75%)	24 (72.7%)
HER2/neu+	2 (12.5%)	6 (18.2%)
Triple Negative	2 (12.5%)	3 (9.1%)
Treatment Characteristics		
Surgery		
Mastectomy	15 (93.8%)	21 (63.6%)
Lumpectomy	1 (6.3%)	12 (36.4%)
Chemotherapy		
Neoadjuvant	6 (37.5%)	14 (42.4%)
Adjuvant	10 (67.5%)	12 (36.4%)
None	0 (0%)	7 (21.2%)
Radiotherapy Coverage		
	BR + SCF + IMN: 14 (87.5%)	BR + SCF: 12 (36%)
	CW + SCF + IMN: 1 (6.3%)	CW + SCF: 17 (52%)
	TE + SCF + IMN: 1 (6.3%)	TE + SCF: 4 (12%)
Median Dose (Range), Gy		
IMN	50.4 (50.4)	N/A
SCF	50.4 (45–50.4)	50.4 (45–50.4)
BR/CW/TE	50.4 (50.4)	50.4 (50–50.4)

*Abbreviations*: *BR* breast, *CW* chest wall, *SCF* supraclavicular fossa, *IMN* internal mammary nodes, *TE* tissue expander, *ER* estrogen receptor, *PR* progesterone receptor

Details regarding surgical, systemic, and radiation treatments are summarized in Table 1. Both groups received a median dose of 50.4 Gy to the primary tumor bed and SCF (range, 50–50.4 Gy). For those who received IMN irradiation, median dose was similarly 50.4 Gy (range, 50.4 Gy). Use of mastectomy and chemotherapy was higher in IMN treated group compared to untreated – 93.8% versus 63.6 and 100% versus 78.8%, respectively.

**Fig. 1:**
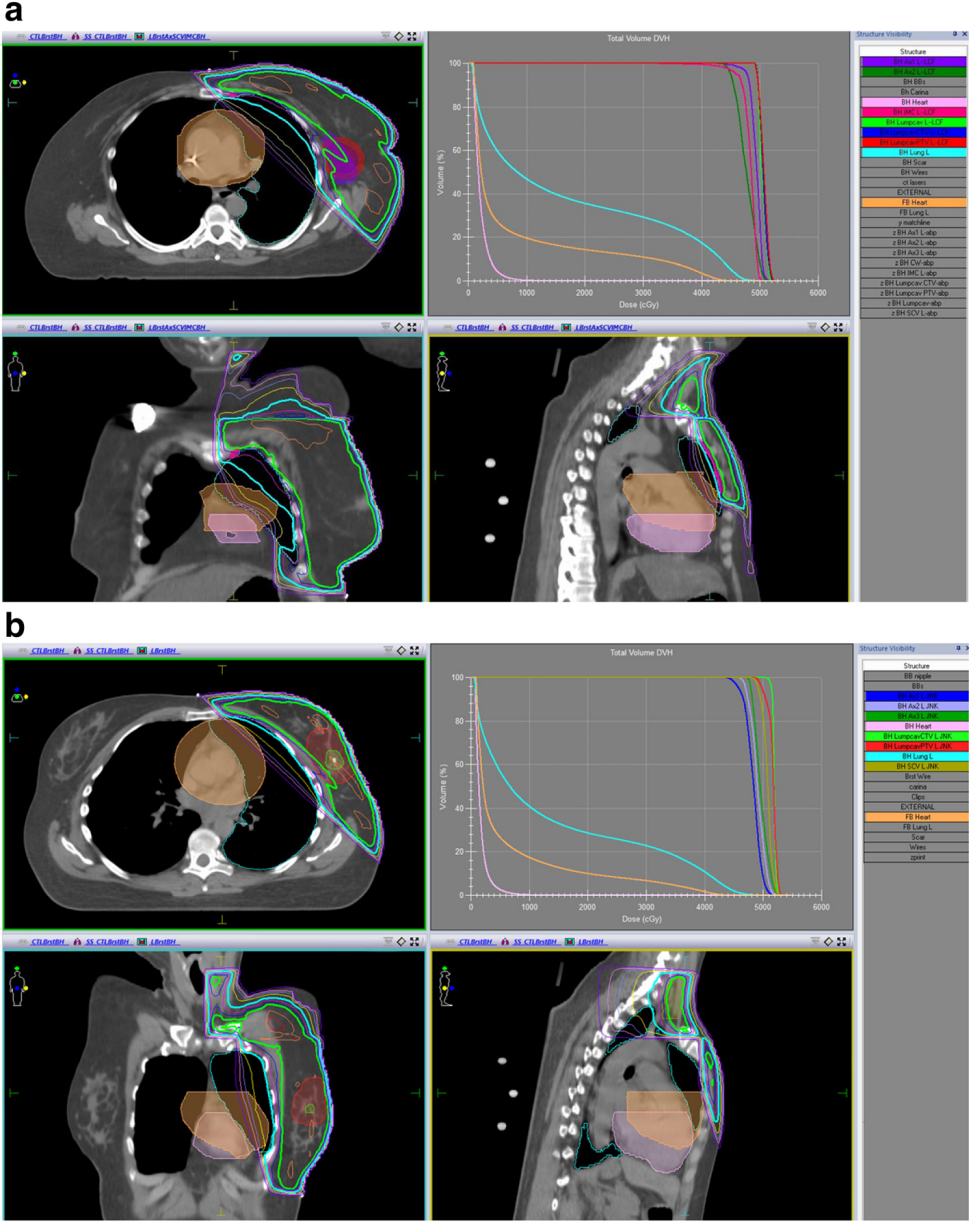
DIBH (BH) dose distribution and FB heart displacement in an **a** IMN treated patient and **b** IMN untreated patient

DIBH significantly reduced the average mean heart dose from 6.73 Gy to 2.79 Gy in the IMN treated group and from 4.77 Gy to 1.55 Gy in the IMN untreated group (both *p* < 0.001). Figure 1 shows a representative dose distribution of a DIBH plan with demonstration of FB heart displacement in a patient from either treatment group. Dose volume measures showed significant decreases in both cohorts at all levels recorded, from V_5_ (relative cardiac volume receiving ≥5 Gy) through V_25_ (all *p* < 0.001), as shown in Table 2. IMN treated patients consistently had a greater extent of cardiac exposure than their untreated peers across all dose volume measures for DIBH (*p* ≤ 0.008) and FB plans (*p* ≤ 0.038) aside from FB V_25_ (*p* = 0.130).

**Table 2:** Comparison of FB versus DIBH mean heart measures within treatment groups

	IMN treated		IMN untreated	
	Absolute value (SD)	*P*-Value	Absolute value (SD)	*P*-Value
Mean Heart Dose (Gy)				
FB	6.73 (1.98)	< 0.001	4.77 (2.44)	< 0.001
DIBH	2.79 (1.23)		1.55 (0.65)	
V_5_ (%)				
FB	34.5 (12.9)	< 0.001	19.4 (9.7)	< 0.001
DIBH	12.7 (10.1)		2.8 (3.4)	
V_10_ (%)				
FB	19.9 (7.4)	< 0.001	11.6 (6.8)	< 0.001
DIBH	4.1 (4.9)		0.8 (1.3)	
V_15_ (%)				
FB	13 (5.7)	< 0.001	8.3 (5.8)	< 0.001
DIBH	2.0 (2.7)		0.4 (0.8)	
V_20_ (%)				
FB	8.9 (4.9)	< 0.001	6.3 (5.2)	< 0.001
DIBH	1.0 (1.5)		0.2 (0.6)	
V_25_ (%)				
FB	6.4 (4.3)	< 0.001	5.0 (4.8)	< 0.001
DIBH	0.4 (0.8)		0.1 (0.5)	

**Fig. 2:**
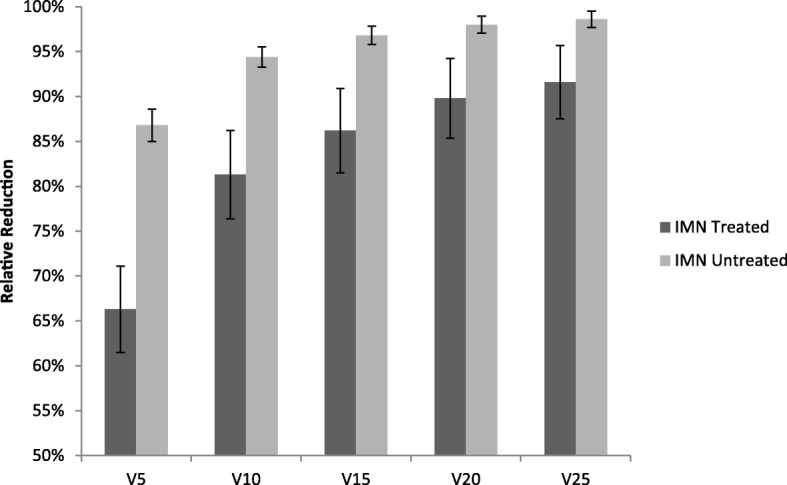
Comparison of mean relative reductions in heart V_5_-V_25_ between treatment groups. All differences were statistically significant (*p* ≤ 0.012)

Relative reductions in mean heart dose and dose volume metrics for both patient groups are shown in Table 3. DIBH allowed for a 56.4% reduction in mean heart dose in IMN treated patients compared to 63.7% in the untreated group, a discrepancy of 7.3% that was not statistically significant (*p* = 0.216). Relative reductions of V_5_ through V_25_ were all significantly lower for IMN irradiated patients (*p* ≤ 0.012), with the greatest separation in relative heart sparing at low doses (V_5_, difference = − 20.5%, *p* < 0.001) that gradually lessened with increasing dose (V_25_, difference = − 7.0%, *p* = 0.007), as represented in Fig. 2.

**Table 3:** Comparison of mean relative reductions in heart measures due to DIBH between treatment groups

	IMN treated (SD), %	IMN untreated (SD), %	Difference, %	*P*-Value
Mean Heart Dose	56.4 (18.4)	63.7 (14.9)	−7.3	0.216
V_5_	66.3 (19.3)	86.8 (10.3)	−20.5	< 0.001
V_10_	81.3 (19.7)	94.4 (6.6)	−13.1	0.007
V_15_	86.2 (18.8)	96.8 (5.8)	−10.6	0.004
V_20_	89.8 (17.7)	98.0 (5.5)	−8.2	0.012
V_25_	91.6 (16.4)	98.6 (5.3)	−7.0	0.007

## Discussion

A multitude of studies have shown that DIBH confers significant reductions in heart exposure in breast radiotherapy, with or without coverage of the draining lymph node basins [20–25]. In one cohort, DIBH was shown to provide greater relative decreases in mean heart dose for patients receiving regional nodal irradiation compared to those who were treated to the breast alone [22], presumably due to innately greater heart exposures associated with nodal irradiation in FB plans. In studies where locoregional radiation has included the IMN, DIBH has consistently reduced both mean heart dose and dose volumes, with preservation of PTV dose coverage [20, 22–26]. However, there remains a paucity of information in regards to the dosimetric properties of IMN treatment itself, apart from other regional nodal groups.

Our results demonstrate that even with the addition of IMN irradiation, the relative benefits of DIBH in reducing mean heart dose are retained. Significant decreases in both cardiac dose and dose volume measures were observed in all patients. While IMN coverage appears to incur small deficits in the relative reductions in V_5_ through V_25_, the heart sparing benefits of DIBH were nevertheless most conserved at the highest doses. Additionally, patients receiving IMN treatment intrinsically begin with a higher heart dose compared to their untreated peers, a difference that is significantly diminished but not erased by the use of the DIBH technique. Large retrospective studies of both breast cancer and Hodgkin lymphoma survivors have estimated an exquisitely similar 7.4% proportional increase in the risk of clinically manifest coronary artery disease per each Gray increase in mean heart dose, irrespective of cardiovascular risks at the time of treatment [27, 28]. Although these studies drew from heterogeneous radiotherapies that spanned multiple decades of care, their conclusions are nevertheless one proposed metric to contextualize the risks and benefits of treatment [29]. In this study, IMN treatment had a higher average mean heart dose (2.79 Gy versus 1.55 Gy) even with DIBH, suggesting that the addition of IMN coverage is associated with an estimated 9.2% proportional increase in the risk of a subsequent major coronary event, based on the conversion factor previously mentioned.

In breast radiotherapy, one of the goals of treatment is to minimize heart exposure to the greatest extent while preserving treatment of target tissues. Historically, women who underwent breast conservation therapy that included radiation showed increased rates of cardiac morbidity and deaths in left-sided patients through 20 years post-treatment [30]. Though many have demonstrated an increased long-term rate of cardiac mortality in the years following radiotherapy, especially for patients with left-sided disease, modern techniques have significantly reduced heart exposure and there is little evidence to support similar degrees of cardiac toxicity in recent decades [31–35]. An extensive review of the National Cancer Institute’s Surveillance, Epidemiology, and End Results (SEER) database on non-metastatic breast cancers diagnosed between 1986 and 1993 found no significant differences in ischemic heart disease, valvular heart disease, conduction abnormalities, or heart failure associated with left-sided radiotherapy compared to right-sided treatment [36]. While these results did not specifically evaluate for the effect of regional nodal irradiation and cannot rule out long-term side effects, they support that heart sparing techniques have greatly diminished the historic risks of laterality.

Recent studies have demonstrated that radiotherapy to regional lymph node basins including the IMN improves disease-free survival and breast cancer mortality [17, 18, 37]. A meta-analysis of three major randomized trials evaluating the effect of regional irradiation revealed that treatment significantly improved disease-free survival (HR 0.86), distant metastasis-free survival (HR 0.84), and overall survival (HR 0.90) [16]. In patients with unilateral early stage node-positive breast cancer, IMN irradiation is associated with small but significant improvements in long-term cancer-specific survival and overall survival [19]. With strong evidence to support potential benefits to disease-free survival, breast cancer mortality, and overall survival, IMN treatment should be thoughtfully considered for early-stage breast cancers. In patients with known cardiovascular risk factors [27], patient-provider discussions should carefully highlight the risks and benefits of comprehensive nodal irradiation.

This study has some limitations including a relatively small sample size and a retrospective single-center design. While based on the experiences at one institution, consecutive patient eligibility and strict selection criteria may lend our results more generalizability to other populations receiving left-breast radiotherapy that includes the IMN. Other cohorts and multicenter studies may be needed to further elucidate our findings.

## Conclusions

Breath hold that is coupled to an accurate and reproducible respiratory monitoring system provides a greatly reduced level of heart exposure, even with the inclusion of the IMN. While dose volume measures experienced small decreases in those who received IMN treatment, the overall benefit of the DIBH technique in diminishing mean heart dose was retained. With the use of modern heart sparing techniques at this institution, left-sided IMN irradiation was associated with an estimated 9.2% proportional increase in the rate of a major coronary event in the years following radiotherapy. However, reported benefits to locoregional control and survival may outweigh these risks. Clinicians should take into account these considerations to help guide when IMN treatment is warranted.

## Abbreviations

BR: Breast
CW: Chest wall
DIBH: Deep inspiration breath hold
ER: Estrogen receptor
FB: Free breathing
IMN: Internal mammary nodes
PR: Progesterone receptor
SCF: Supraclavicular fossa
TE: Tissue expander
